# Feasibility, Efficacy, and Efficiency of eHealth-Supported Pediatric Asthma Care: Six-Month Quasi-Experimental Single-Arm Pretest-Posttest Study

**DOI:** 10.2196/24634

**Published:** 2021-07-26

**Authors:** Mattienne van der Kamp, Pamela Reimering Hartgerink, Jean Driessen, Bernard Thio, Hermie Hermens, Monique Tabak

**Affiliations:** 1 Department of Pediatrics Medisch Spectrum Twente Enschede Netherlands; 2 Biomedical Signals and Systems University of Twente Enschede Netherlands; 3 Department of Sports Medicine Orthopedisch Centrum Oost Nederland Hengelo Netherlands; 4 Department of eHealth Roessingh Research and Development Enschede Netherlands

**Keywords:** telemedicine, feasibility studies, child, self-management, asthma, patient acceptance of health care, ambulatory care, remote sensing technology, cost-benefit analysis, health care costs

## Abstract

**Background:**

Early detection of loss of asthma control can effectively reduce the burden of the disease. However, broad implementation in clinical practice has not been accomplished so far. We are in need of research investigating the operationalization of eHealth pediatric asthma care in practice, which can provide the most potential benefits in terms of adoption, efficiency, and effectiveness.

**Objective:**

The aim of this study was to investigate the technical and clinical feasibility, including an exploration of the efficacy and cost-efficiency, of an eHealth program implemented in daily clinical pediatric asthma practice.

**Methods:**

We designed an eHealth-supported pediatric asthma program facilitating early detection of loss of asthma control while increasing symptom awareness and self-management. In the 6-month program, asthma control was monitored by 4 health care professionals (HCPs) by using objective home measurements and the web-based Puffer app to allow timely medical anticipation and prevent treatment delay. Technical feasibility was assessed by technology use, system usability, and technology acceptance. Clinical feasibility was assessed by participation and patient-reported health and care outcomes and via a focus group with HCPs regarding their experiences of implementing eHealth in daily practice. The efficacy and cost-efficiency were explored by comparing pretest-posttest program differences in asthma outcomes (asthma control, lung function, and therapy adherence) and medical consumption.

**Results:**

Of 41 children, 35 children with moderate-to-severe asthma volunteered for participation. With regard to technical feasibility, the Puffer app scored a good usability score of 78 on the System Usability Scale and a score of 70 for technology acceptance on a scale of 1 to 100. Approximately 75% (18/24) of the children indicated that eHealth helped them to control their asthma during the program. HCPs indicated that home measurements and real time communication enabled them to make safe and substantiated medical decisions during symptom manifestations. With an average time commitment of 15 minutes by patients, eHealth care led to a 80% gross reduction (from €71,784 to €14,018, US $1=€0.85) in health care utilization, 8.6% increase (from 18.6 to 20.2, *P*=.40) in asthma control, 25.0% increase (from 2.8 to 3.5, *P*=.04) in the self-management level, and 20.4% improved (from 71.2 to 76.8, *P*=.02) therapy adherence.

**Conclusions:**

eHealth asthma care seems to be technically and clinically feasible, enables safe remote care, and seems to be beneficial for pediatric asthma care in terms of health outcomes and health care utilization. Follow-up research should focus on targeted effectiveness studies with the lessons learned, while also enabling individualization of eHealth for personalized health care.

## Introduction

Asthma is one of the most common chronic diseases in childhood with an estimated prevalence of 7%-10% [1]. Pediatric asthma is an episodic obstructive airway disease leading to attacks, which can hamper physical and emotional well-being [2-4]. The organization of long-term pediatric asthma care currently consists of scheduled periodical hospital visits during which children are clinically evaluated, while being usually symptom-free [5]. Parents and children are also educated during these visits to recognize loss of asthma control and instructed how to manage their symptoms when they occur at home. This, however, relies on an accurate symptom perception of parents and children, which is inaccurate one-third of the times [6,7]. Sears et al [8] showed that inadequate assessment of severity and failure of the family to call for help when required are the major risk factors for serious exacerbations and deaths due to pediatric asthma. Asthma attacks are still one of the main causes of emergency department visits and hospitalizations, thereby imposing a great burden on the pediatric health care system [9-11]. 

Previous studies have shown that the implementation of strategies aimed at the early detection of asthma, thereby providing access to proper and timely treatment, effectively reduced the burden of the disease [5,9,12]. However, these strategies have not been implemented on a large scale in clinical practice [13]. Health care professionals (HCPs), children, and parents may lack reliable and affordable tools, which can unobtrusively assist disease monitoring and improve health outcomes. eHealth pediatric asthma care supported by home-monitoring technology such as hand-held spirometers or smart inhalers could be such a strategy as it can provide (1) quantitative insight into the dynamics of chronic disease progression; (2) insight into the severity, dynamics, and perception of asthma symptoms, as it exploits repeated measurements of asthma status during symptomatic periods, thereby enabling self-assessment and self-management [14,15] and building symptom perception [16]; and (3) early detection of loss of control and identification of cues and causes of asthma control deterioration [17], which could facilitate timely and targeted medical anticipation and rapid regain of the control of asthma, preventing asthma attacks. Combining these aspects, eHealth strategies may optimize and increase compliance to treatment regimens and may be beneficial in improving health outcomes [18].

Existing evidence on the impact of eHealth in the management of asthma has high heterogeneity in the study endpoints and designs [19]. To date, the largest proportion of eHealth research zeros in on either improving therapy adherence [20-22] or boosting self-management [23-25] and is often not specifically tailored to the pediatric population. The research gap, therefore, lies in the development and evaluation of an eHealth strategy for children with asthma that is based on real-time communication with HCPs and a multi-parameter monitoring approach that facilitates timely anticipation in case of worsening of disease progression. Reaching optimal effects of eHealth care is conditional upon the (1) readiness, acceptance, and engagement of the technology, (2) reliability of the clinical content, and (3) adoption and implementation in clinical practice [26-29]. Only if these 3 conditions are met, optimal efficacy can be expected and options for permanent embedding in practice can be properly evaluated. Currently, the evaluations of eHealth interventions are often executed within either a short pilot or a larger controlled research setting, both lacking to fulfil condition 3 (adoption and implementation in clinical practice) and therefore, not resembling daily care practice. Many of these studies indicate that more research is needed to evaluate the barriers and facilitators for the implementation of an eHealth program in daily practice outside a study setting [30]. Therefore, in this study, we used an exploratory study design adopted in daily clinical care to investigate the operationalization of an eHealth pediatric asthma care program, supported by home-monitoring technology. This study investigates the technical feasibility, that is, technology use, usability, and acceptance, and clinical feasibility, that is, where we can expect the greatest effects and under what conditions we can expect these. The clinical feasibility includes an exploration of the efficacy (in terms of self-reported asthma outcomes, therapy adherence, and inhalation technique combined with lung function) and cost-efficiency (ie, health care utilization). The lessons learned from this study can lay the foundation for targeted effectiveness studies [30].

## Methods

### Study Design

This exploratory study had a quasi-experiment single arm pretest-posttest design to assess the feasibility of an eHealth program implemented in pediatric asthma care. To explore the efficacy and efficiency of the eHealth program compared to those of regular care, historical data were used for comparison.

### Subjects

In total, 41 children (age 4-18 years) with moderate-to-severe pediatrician-diagnosed asthma were asked to participate. They were recruited from the pediatric department of Medisch Spectrum Twente, Enschede, The Netherlands between July 2018 and May 2019 by using consecutive sampling. Children with comorbid chronic diseases or children/parents unable to understand or speak Dutch were not eligible to participate. Offline written informed consent from parents and children >12 years was obtained prior to enrolment. During the exploratory eHealth program, both the HCPs and children and parents could restore regular outpatient follow-up if desired or medically justified.

### The eHealth Program

The eHealth program (Multimedia Appendix 1) was designed to detect the loss of asthma control in daily life timely and accurately [17], to increase awareness of the severity of asthma symptoms, and to improve the safety of care for both physicians and patients by using objective measurements as the basis for joint decision making. The development and content of the eHealth program was frozen during this study and consisted of the Puffer app and a set of 2 monitoring devices:

1. Monitoring of lung function was performed using the hand-held Spirobank advanced II (MIR Inc). Spirometer flow-volume loops were classified by the HCPs based on self-reported events (regular, pre-exercise, postexercise, symptom, after reliever use). Incorrectly performed spirometer measurements were excluded, according to the American Thoracic Society and European Thoracic Society criteria for standardization of lung function testing [31]. Single spirometry outcome measures (such as forced expiratory volume in 1 second [FEV_1_], FEV_1_/forced vital capacity [FVC], FEF_25-75_ [mean forced expiratory flow between 25% and 75% of the FVC], and peak expiratory flow) and combined measures (pre-post exercise FEV_1_ differences, pre-post reliever use FEV_1_ differences and FEV_1_ variation) were monitored. Children were asked to perform a spirometry measurement once a week and during symptom occurrence.
2. Medication adherence and inhalation techniques were electronically tracked with the Amiko Respiro smart inhalers (Amiko Inc). This information is essential as many studies have shown that adherence and inhaler technique in children with asthma is poor [32,33]. Moreover, Chrystyn et al [34] recently indicated that smart inhaler studies need to be carried out to demonstrate their potential to improve disease control, prevent exacerbations, and justify their costs. Controller adherence was calculated by dividing the amount of controller medication taken by the amount of medication prescribed (%). Inhalation technique data consisted of the inhalation flow, inhalation duration, and device orientation and were visualized with respect to the regulative values per inhalation device.

**Figure 1:**
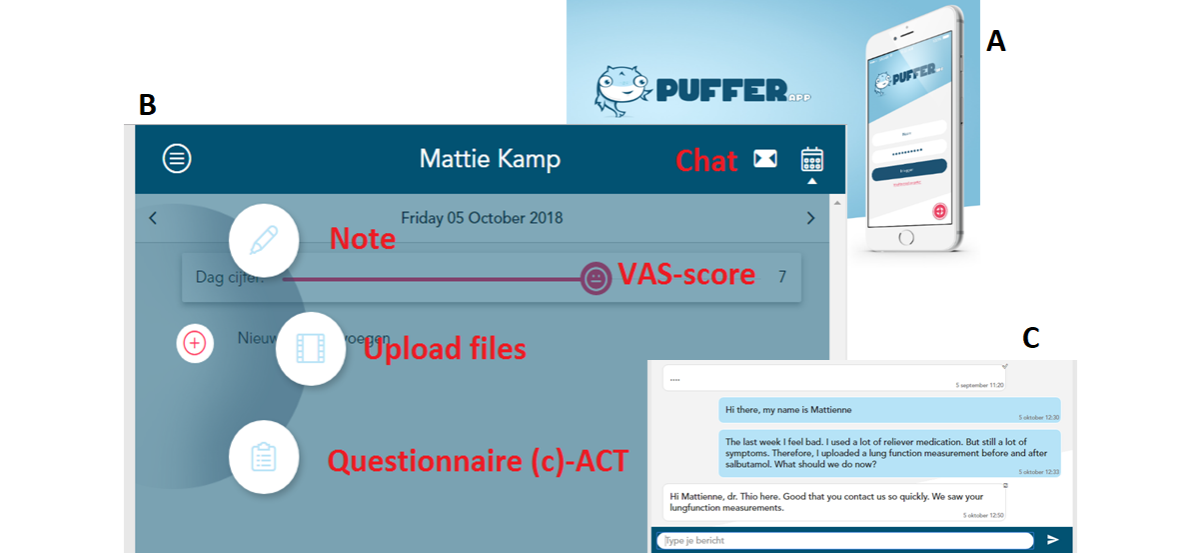
An overview of the Puffer app. A: image of the logo and design of log-in screen with username and password; B: overview of the functionalities (the red words indicate the functionalities); C: chat screen. ACT: asthma control test; VAS: visual analog scale.

The web-based Puffer app (Figure 1) consists of the following functionalities:

1. Share photos, videos, and sound recordings: Sharing symptom recordings (ie, allergic reactions, wheeze, cough, and dyspnea) allowed the assessment of asthma severity, especially in younger children [35]. These children have little capacity to compensate for hypoxia and a compliant chest wall; therefore, videos may reveal multiple observational signs (eg, tachypnea, retractions, nasal flaring, speaking in words) [36]. The video option could also be used, as needed, to assess the adequate use of the inhaler and spirometer [37].
2. Chat function: The chats were stamped with a date and time label and the messages receive a checkmark when read by the HCPs and vice versa.
3. Emergency button: It provides the emergency action plan.
4. Share monitoring data: Children could indicate their daily dyspnea symptoms using the visual analog scale (VAS) [38] indicated by emojis, 1 (worst dyspnea ever: sad crying emoji) and 10 (no dyspnea at all: happy face emoji). Asthma control was monitored via the childhood asthma control test (C-ACT) questionnaire [39].

The program was offered for 6 months. Before start, children and parents received the monitoring devices and app, instruction materials, and were instructed on site by the HCPs. A self-made animation (Multimedia Appendix 2) was shared with the participants to illustrate the purpose of the program. Participants were in contact from home with their HCPs (nurse practitioners, technical physician, and pediatric pulmonologist) via a web-based app (Puffer app). Within the 6-month program period, children and parents were free to use the Puffer app whenever they felt to but were encouraged to update the HCPs at least weekly. Moreover, children and parents were instructed to contact the HCPs as soon as possible when symptoms occur but were also explicitly instructed to not wait for web-based communication in case of emergency asthma exacerbations by pursuing the regular paths within the health care system. HCPs checked the Puffer app daily for new content and data and provided personalized advice via the chat based on the communication and monitored data. All data were visible to both the participants and HCPs. Moreover, once a week, the HCPs came together for a multidisciplinary consultation, in which the data trends and the communications of all patients were discussed.

### Outcome Measures

Demographic characteristics (ie, age, gender, inhaled corticosteroid use, long-acting beta-agonist use, and inhalation allergy) were retrieved from the electronic patient record. The health care utilization of the patient was categorized (light ambulatory, middle ambulatory, and clinical) according to the Dutch healthcare registration system of the Dutch Health Care Authority.

#### Technical Feasibility

Technical feasibility was assessed by technology use, system usability, and technology acceptance. Technology use was determined continuously by the number of chat messages, time spent using the Puffer app (minutes/week), and the adherence (%) of the spirometry data uploads (assuming 1 lung function measurement per week). System usability and technology acceptance were assessed using the System Usability Scale and Technology Acceptance Model [40,41] at the end of the eHealth program (T_end_), and in addition, by means of a nondirective interview (with an average duration of 5 min) in which the children and parents were asked to provide their experiences of using the technology as part of the eHealth program. From the interview, an overview of the issues was made by grouping similar issues and those were converted into categorical codes (0=negative, 1=positive) to allow for statistical analyses. Moreover, the issues were categorized as minor, serious, or critical based on the frequency and consequences as described by Duh et al [42] and verified by the involved HCPs.

#### Clinical Feasibility

Clinical feasibility was assessed by participation rate, patient-reported health and care outcomes, and implementation experiences of the HCPs by exploring efficacy and efficiency. The participation rate was the proportion of children who volunteered to participate after being approached for participation. The patient-reported outcomes were quality of care (client satisfaction questionnaire-8 items [CSQ-8]), self-management level (patient activation measure-13 items questionnaire), and quality of life (EuroQol-5D) [43-45]. In addition, participants were also asked whether the proposed eHealth care could support them to control their asthma and whether it could help to prevent emergency department visits and admissions to the hospital (with the answer options: yes absolutely, I don’t know, and no). All patient-reported outcomes were assessed on paper prior to the start of the eHealth program (T_start_) and at the end of the eHealth program (T_end_). To assess the experiences of HCPs with the eHealth program, 4 HCPs that were part of the eHealth care team were asked to verbalize their thoughts and practical experiences of the eHealth care in a focus group of approximately 60 minutes. The primary aim of the focus group was to identify the barriers and facilitators of pediatric eHealth care, to investigate to what extent the eHealth care program is implementable in their pediatric asthma care center, and to yield pragmatic recommendations. The outcomes were structured under the themes: technical innovations, eHealth asthma care, and implementation considerations [46].

The efficacy of the eHealth care was assessed by lung function tests at home, therapy adherence, inhalation technique, and self-reported asthma outcomes (C-ACT scores and VAS scores of dyspnea) [38,39]. Efficacy was explored by investigating the change in asthma outcomes between the start (first 3 measurements within the first month) and end of the eHealth care (last 3 measurements within the last month of the eHealth care). Health care utilization data consisted of all asthma-related medical procedures (diagnostics, therapy, admissions, emergency department visits, consultations) and were retrieved from the hospital registration system. The unit cost prices of these procedures were determined according to the cost price model guideline of the Dutch Health Care Authority [47]. The costs of the eHealth care program were evaluated by combining the depreciation costs of the equipment and the additional workhours of all HCPs. Research-related costs were excluded. The efficiency of the care was then explored by a within-subjects paired comparison between (1) the historical health care utilization data from a half year prior to the inclusion till the moment of inclusion and (2) the health care utilization data during the eHealth program.

### Statistical Analysis

This explorative study used a per-protocol analysis as the dropout rate was low, causing the analysis to better reflect the effects of eHealth when used adherently and without complications in the majority of the asthmatic children. Missing data of children who finished the eHealth program were handled by pairwise deletion. Descriptive statistics were used to examine all the continuous outcome measures and were expressed in mean (SD) for normally distributed variables and in median (IQR) for not normally distributed variables. Univariate analyses were performed on the pretest-posttest differences of the patient-reported outcomes, asthma outcomes, and care utilization with SPSS statistics (IBM Corp). The Shapiro-Wilk test was used to determine whether the variables were normally distributed. The variables that did not have a normal distribution were tested for paired differences with the Wilcoxon signed-rank test. Normally distributed variables were tested with a paired two-tailed *t* test. *P* values less than or equal to .05 were considered as significant.

## Results

### Demographic Characteristics

Of the 35 children who participated, 30 children (mean age 11.1 [SD 4.1] years, 22 boys) finished the half-year eHealth care period. Table 1 shows an overview of the characteristics of the children. The majority (25/30) of these children had a high health care utilization.

**Table 1:** Patient characteristics (n=30).

Patient characteristics		Value
Age (years), mean (SD)		11.1 (4.1)
Gender (male), n (%)		22 (73)
BMI z-score, mean (SD)		0.52 (0.87)
Inhaled corticosteroid use, n (%)		30 (100)
Long-acting beta-agonist use, n (%)		24 (80)
Inhalation allergy, n (%)		27 (90)
Childhood asthma control test score, mean (SD)		18.6 (5.0)
**Asthma care registration (in half year prior to inclusion), n (%)**		
	Light ambulatory^a^	5 (17)
	Middle ambulatory^b^	15 (50)
	Clinical^c^	10 (33)

^a^Light ambulatory is defined as having 1 or 2 outpatient visits for pediatric asthma without additional care utility.

^b^Middle ambulatory is defined as having 3 or more outpatient visits or day treatment or diagnostic testing or any combination of these.

^c^Clinical is defined as having a hospital admission for pediatric asthma.

### Technical Feasibility

With regard to technology use, on average, 103 (SD 71) chat messages were sent and received per patient within the half year eHealth care, which is approximately 2 messages per patient per week. The median time spent on using the Puffer app by the participants was 15 minutes per week (IQR 10-26.25 minutes per week). The spirometry adherence was on average 55.7% (SD 9.5%), with week 4 as their median first week for skipping the data upload and week 10 as their median time to have skipped 3 weeks of spirometry data. The system usability score was 78 (SD 17), which indicated good usability. The technology acceptance score was 70 (SD 18) on a scale from 0 to 100. The specific components that made up the technology acceptance showed the highest scores on intention to use (81 [SD 17%]) and ease of use (77 [SD 24%]) and lowest on control over the system (64 [SD 25%]). No critical issues were identified from the nondirective interview with children and parents. Six participants indicated the serious issue that “it would be useful if you were notified if there was a new message from the doctor,” 3 participants preferred a native app instead of a web-based app, and 2 participants indicated that they would like a graphical overview for inspection of their own data.

### Clinical Feasibility

Participation rate was high, as 85% (35/41) of the eligible children were willing to participate in the eHealth program. Of these 35 children, 2 dropped out as they indicated to prefer regular care because the eHealth care required “too much effort” from their side. The other 3 excluded participants were prematurely excluded on indication of the HCPs as they felt that the responsibility of care came at stake owing to insufficient good quality home-monitoring data, repeated technology issues that could not be solved remotely, or late responses of the participants to symptoms. Table 2 shows the patient-reported health and care outcomes at the start and at the end of the eHealth program. It reveals that the self-management score and the associated self-management level (patient activation measure-13 item questionnaire) significantly increased through participation in the eHealth care program (*P*=.02). The quality of care (CSQ-8) was significantly lower at the end of the program (*P*=.03). The quality of life showed no significant paired differences. Prior to the start of the project, 77% (10/13) of the participants indicated that eHealth could help to control the disease compared to 75% (18/24) afterwards. Prior to the start of the project, 69% (9/13) of the participants indicated that eHealth could help to prevent admissions and emergency department visits compared to 92% (22/24) afterwards.

The focus group was attended by 4 HCPs (a pediatric pulmonologist, a technical physician, a nurse practitioner, and an asthma nurse), who were all part of the eHealth asthma team. Table 3 shows an overview of the barriers, facilitators, and recommendations mentioned, categorized into 3 main domains: technical innovations, eHealth asthma care, and implementation considerations. An elaborate written collection of the experiences of the HCPs can be found in Multimedia Appendix 3.

**Table 2:** Patient-reported outcomes.

Outcome			Patients (n)		Start of eHealth care intervention, mean (SD)		End of eHealth care intervention, mean (SD)		Relative difference (%)		*P* value
Quality of care (client satisfaction questionnaire-8 items) (0%-100%)			9		94.4 (4.9)		84.7 (10.6)		–10		.03
**Self-management score**											
	Patient activation measure-13 items (13-52)	10		40.8 (4.3)		44.2 (5.0)		+8		.02	
	Patient activation measure level^a^ (1-4)	10		2.8 (0.9)		3.5 (0.7)		+25		.04	
	Quality of life (EuroQol-5D, 0-100)^b^	10		94.8 (8.4)		93.0 (10.8)		–2		.50	

^a^Level 1: start taking on a role, level 2: building knowledge and trust, level 3: take action, level 4: sustain behavior.

^b^EuroQol-5D: European Quality of Life-5 dimension scale.

**Table 3:** An overview of the barriers, facilitators, and recommendations of the health care professionals.

Theme, subtheme		Barriers	Facilitators	Recommendations
**Technical innovations**				
	General	Technical difficulties for children/parents learning to operate new diagnostic devices	Objective assessment Visualization of trend data	Create help desk Expand instruction with run-through on own devices
	Smart inhaler	Nuisance of device updates Limited compatibility to iPhone operating system Limited number of inhalers compatible	Ability to track therapy compliance real-time Gain insight into medication use behavior Assessing inhalation technique to select appropriate inhaler type for child	Expand range of compatible (pediatric) inhalers Automatic synchronization
	Puffer app	No integration with electronic health record Absence of real-time reminding system	Video assessment of symptoms Assessment of symptom perception by combining subjective and objective measures	Include pop-up reminders Connect data trend log to electronic health record
**eHealth asthma care**				
	General	Risk of missing symptoms in case of noncompliance to eHealth Lack of physical examination	Individualized care plan Safe “substantiated by data” medical decision making Ability to step-wise learn for children/parents to self-manage asthma Shared care responsibility between health care professionals and children/parents	Children with uncontrolled moderate-to-severe asthma are primarily suited for eHealth Discuss the overlapping disease management goals at start of eHealth period The extent of eHealth care should be adaptable and confined to the individual needs.
**Implementation considerations**				
	Time investment	Difficult to schedule time for eHealth care due to its varying character Increased time expenditure per patient	Parents/children admire the additional time effort	Regional cooperation to enable scheduled shifts No fixed eHealth period; option to quickly de-escalate eHealth care and option to easily restart.
	Health care professionals	Requires reorganization of personnel	Efficient task reallocation Multidisciplinary approach	Weekly multidisciplinary consultation Include a technical oriented care professional to the eHealth team.
	Compliance	Less compliance to eHealth in times when symptoms are not perceived	Ability to automatically track compliance to care tasks	Create a weekly routine of measurements Transparent noncompliance flowchart to protocolize reminders and eventual exclusion

### Efficacy Outcomes

This eHealth program led to an improvement in asthma outcomes, as shown in Table 4. It is noticeable that lung function (+10%), self-interpreted dyspnea (VAS) (+8%), and therapy compliance (+20%) increased significantly. Moreover, an average increase of 1.7 points in the C-ACT score was noticeable after eHealth care, shifting the average from uncontrolled asthma (≤19) to controlled asthma (>20); however, this difference was not statistically significant.

**Table 4:** Asthma outcomes.

Outcome measure	Patients (n)	Start of eHealth care, mean (SD)	End of eHealth care, mean (SD)	Relative difference (%)	*P* value
Lung function (forced expiratory volume in 1 second % predicted)	24	82.2% (18.1)	90.1 (18.1)	+10	<.001
Dyspnea score (visual analog scale 1-10)^a^	17	7.8 (1.5)	8.4 (1.0)	+8	.01
Childhood asthma control test	9	18.6 (5.0)	20.2 (4.0)	+9	.40
Therapy adherence (% of prescribed)	16	59.9 (33.3)	72.1 (20.3)	+20	.02
Inhalation technique (% correct intake)	16	71.2 (27.4)	76.8 (18.9)	+8	.09

^a^Score1: most severe imaginable dyspnea symptoms; score 10: no symptoms of dyspnea at all.

### Efficiency Outcomes

The asthma care registration before and after eHealth care changed as follows: clinical T_start_, n=10, T_end_, n=2; middle ambulatory T_start_, n=15, T_end_, n=2; and light ambulatory T_start_, n=5, T_end_, n=26. Moreover, Table 5 shows that eHealth care resulted in a reduction of care utilization in all aspects, with 85% (from 13 to 2 admissions, 11/13) fewer hospital admissions, 81% (from 21 to 4 emergency visits, 17/21) fewer emergency department visits, and 83% (from 116 to 20 outpatient visits, 96/116) fewer outpatient visits. The reduction in care utilization ensured an average cost reduction per patient of €1925.52 (US $1=€0.85) per half year (80%). Of this, 38.2% (735.55/1925.52) is covered by outpatient savings and therefore 61.8% (1189.97/1925.52) by savings on clinical care. The average program cost was €1291.50 consisting of €420 for the monitoring devices and €871.50 for the additional hours of the HCPs. Therefore, the net cost reduction was 26.3% (634.02/1925.52).

**Table 5:** Health care utilization.

Care utilization	Six months prior to inclusion	Six months in eHealth care	Difference (relative difference in %)
Hospital admissions (n)	13	2	–11 (–85)
Emergency visits (n)	21	4	–17 (–81)
Outpatient visits (n)	116	20	–96 (–83)
Diagnostic tests (n)	20	1	–19 (–95)
Telephonic consultation (n)	21	9	–12 (–57)
Total health care costs (euro)^a^	€71,784	€14,018	–€57,766 (–80)
Program costs (euro)	N/A^b^	€38,745	+€38,745
Net cost reduction	€71,784	€52,763	–€19,021 (–26)

^a^US $1=€0.85.

^b^N/A: not applicable.

## Discussion

### Principal Findings

This exploratory study revealed a high feasibility for the use of eHealth-supported pediatric asthma care to monitor and manage children with moderate-to-severe asthma. The exploratory findings showed an increase in self-management, lung function, and therapy adherence and a gross reduction in health care utilization of 80% compared to the historical medical utilization in the same patients. With regard to the technical feasibility, the eHealth system showed good usability and good technology acceptance. No critical issues were identified, but improvements were suggested by patients and HCPs to increase compatibility, enable reminders, and work toward a higher technology readiness level. The technology use of participants was sufficient but became less adherent over time and were mainly adherent to the instructed monitoring frequency in periods with increasing asthma symptoms, consistent with the “law of attraction” as previously described by Eysenbach [48] and comparable to other asthma telehealth tools [49]. Participants indicated that the lack of time and lack of pop-up reminders made them forget to share data/communications. Moreover, participants may grow into “e-attainers,” thereby receiving what was needed (eg, experienced symptom reduction) from this program, even if not from the HCPs, and they would become less adherent [13,50].

This study showed that eHealth-supported asthma care can be beneficial for patients, HCPs, and care organizations. With a participation rate of 85.4%, our eHealth program compared well to other pediatric eHealth initiatives, especially considering the half year time span of the study [51-53]. The high willingness to participate in eHealth care could be due to strengthened position of the children and parents by engaging in their own health care [54]. This provided an opportunity for them to express themselves, measure their symptoms, discuss their insecurities, and enabled them to participate in decision making [55]. Moreover, 75% of the participating children/parents were convinced that this type of eHealth care could help control the disease. In line with this, our study revealed a significant increase in self-management, which is in line with the meta-analytic review of Cushing and Steele [56] who stated, “eHealth interventions that incorporate behavioral methods (eg, self-monitoring, goal setting, immediate feedback, contingency management) produce larger effect sizes for health behaviors and their associated outcomes than interventions that rely solely on education.” Moreover, de Jongh et al [57] reported that mobile phone messaging may facilitate self-management of long-term illnesses, emphasizing the importance of direct communication between HCPs and patients. In particular, communication substantiated by self-monitoring data can build up the confidence of children and parents and enhance understanding and self-management of disease. The C-ACT score did not show a significant improvement. However, the average increase from 18.6 (which reflects uncontrolled asthma) to 20.2 (which is clinically interpreted as controlled asthma) indicates a fair margin of improvement in the moderate-to-severe asthma in the population included in this study [5].

This study showed a 80% gross reduction in health care utilization, which was also reflected by the beliefs of the participants themselves; 77% (23/30) of the parents at the start of the program and 92% (24/26) after participating in the program claimed that eHealth in children with asthma could help prevent admissions and emergency department visits. The eHealth care provided a platform for transparent knowledge transfer about the course of individual asthma symptoms and how to manage these. This may have undermined a common belief in patients that asthma is an acute rather than a chronic condition [58], leading to improved therapy compliance and better asthma outcomes in a majority of the children and parents.

### Implications For Future Research and Daily Care Practice

Although most eHealth interventions report improved patient outcomes, there still is skepticism about the use of eHealth [59-61]. What is the balance between obtrusiveness of home measurements versus the relevance for disease monitoring? How does eHealth adapt to the individual needs of a patient? Does continuous data collection at home compete with privacy rights and how are data securely managed, processed, and stored? These aforementioned barriers combined with the contextual obstacles (such as workplace reorganization and changes in employment and work priorities) contribute to the conservative attitude of HCPs toward eHealth [59,60]. In contrast, this proof-of-concept study and the study of Simpson et al [62] show legitimate support of mHealth to assist with asthma self-management by both individuals with asthma and HCPs. The HCPs who participated in this study’s focus group were confident and enthusiastic about the potential of eHealth care and indicated that current barriers in the organizational and technological aspects are solvable. Specific future focus should be on the safety aspects of eHealth care so that noncompliance to eHealth cannot lead to missing crucial disease information. HCPs also expressed that the ever-expanding data-driven community combined with the increasing amount and quality of available eHealth technologies will slowly occupy a permanent place within the pediatric asthma care. Nonetheless, there is still a lot to gain in bringing these interventions to practice. Next to technological and contextual improvements, HCPs indicated that follow-up research should focus on investigating the adoption of eHealth within the medical guidelines, individualization of eHealth interventions, and protocolization of eHealth use for specific subissues (ie, poor adherence, at risk for exacerbations, and low self-management level). Although cost-effectiveness is particularly important in health care, only few eHealth systems have demonstrated economic advantage, which makes investments in technology by commissioners of services unlikely and implementation even harder [61]. This exploratory study investigated the economic effect and revealed a marked reduction in health care costs by secondary and tertiary prevention with the use of objective monitoring and direct communication. Taking into account the task reallocation of the HCPs and the additional costs of eHealth care resulted in an estimated net cost reduction of 26%, enabling further steps for financial coverage.

### Strengths and Limitations

The eHealth program incorporated combined sensing technologies, which could be used to monitor and estimate the disease course, enabling HCPs to anticipate early by medical interventions. This has not been explored in the field of pediatric eHealth care effect studies before and builds upon existing evidence of improved asthma outcomes in eHealth studies using questionnaire-based asthma monitoring or digital self-management support [63-66]. Moreover, our program focused on the development of self-management by using quick, substantiated (by data), and personalized communication by the HCPs in periods of symptoms. Another strength of this program was that children with moderate-to-severe asthma were included and that the baseline characteristics reflect this population well. This population is eminently the group at risk for exacerbations and hospitalizations, and therefore, the first target group for eHealth interventions to enable more effective and efficient pediatric asthma care [67].

This study was limited by the amount of missing questionnaire data. Questionnaires that were not filled completely or incorrectly (multiple answers given in multiple choice) were excluded from the analysis to retain the validity of the questionnaire scores. Moreover, some participants returned the questionnaires by post, which additionally contributed to missing data. Web-based survey systems may help to overcome these issues [68]. The EuroQol-5D quality of life questionnaire showed to be prone to ceiling effects in the pediatric asthma population, as the questions do not really correspond to the burden of asthma on children. Therefore, specific quality-of-life questionnaires for pediatric asthma such as the pediatric asthma quality of life questionnaire are recommended [69,70].

Although this program strongly suggests that eHealth asthma care might enhance asthma outcomes with a reduction in hospital care utilization, this study was not designed as a randomized controlled trial. At the start of the program, the development of the Puffer app was frozen, but the knowledge, skills, and expertise of HCPs for applying eHealth technology in the pediatric asthma care was expected to progress, which made it better suited for a quality improvement methodology [13]. This study, therefore, demands replication and validation with a control group.

### Conclusions

This study revealed a high feasibility for the use of ambulatory pediatric asthma care supported by combined sensing technology that monitors moderate-to-severe asthma and provides timely and substantiated medical anticipation. Future research should focus on investigating adoption of eHealth within the medical pediatric asthma guidelines and individualization of eHealth interventions to reach maximal adoption. These studies can contribute to the development and implementation of feasible ambulatory pediatric asthma interventions, which may help reducing the health burden by increasing long-term respiratory health outcomes.

## Appendix

Multimedia Appendix 1 Flowchart showing the overview of the eHealth program.

Multimedia Appendix 2 eHealth program animation.

Multimedia Appendix 3 Summary document of the focus group: the experiences of the health care professionals.

## Abbreviations

C-ACT: childhood asthma control test
CSQ-8: client satisfaction questionnaire-8 items
FEF: forced expiratory flow
FEV_1_: forced expiratory volume in 1 second
FVC: forced vital capacity
HCP: health care professional
VAS: visual analog scale
