# Supplementary figures and images for: Feasibility, Efficacy, and Efficiency of eHealth-Supported Pediatric Asthma Care: Six-Month Quasi-Experimental Single-Arm Pretest-Posttest Study
Source: JMIR Form Res. 2021 Jul 26;5(7):e24634. doi: 10.2196/24634 (PMC8367169; doi:10.2196/24634)

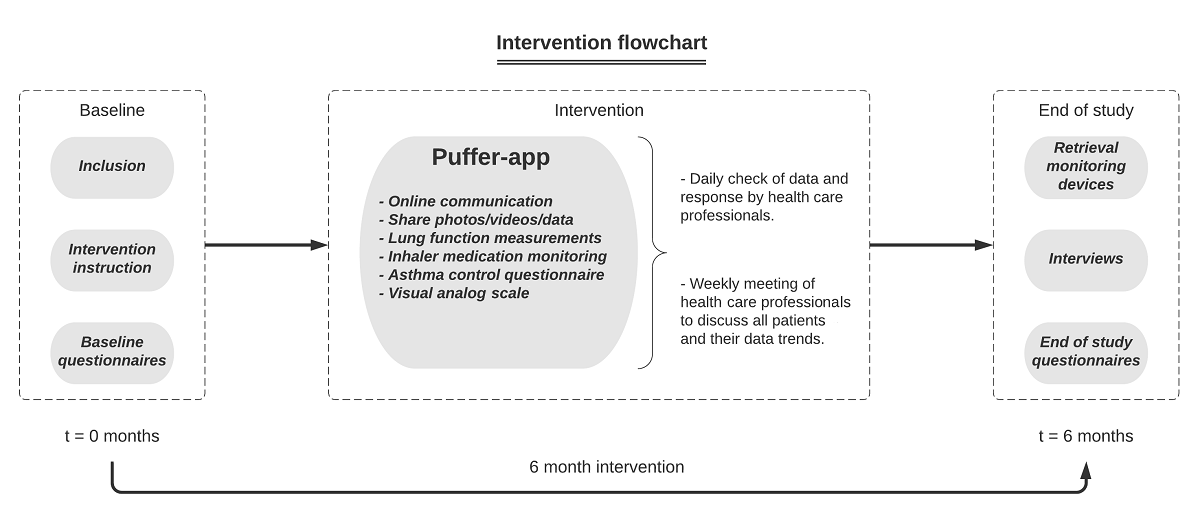

Supplement: Multimedia Appendix 1 [file formative_v5i7e24634_app1.png]
