# Supplementary material for: Feasibility, Efficacy, and Efficiency of eHealth-Supported Pediatric Asthma Care: Six-Month Quasi-Experimental Single-Arm Pretest-Posttest Study
Source: JMIR Form Res. 2021 Jul 26;5(7):e24634. doi: 10.2196/24634 (PMC8367169; doi:10.2196/24634)
Supplement: Multimedia Appendix 3 [file formative_v5i7e24634_app3.docx]

# Supplementary file 3:

### Experiences from the health care professionals.

*Instruction session:* Both the health care professionals (HCPs) and the patients reported that the instruction session was essential for a good start of eHealth period. The nurse practitioner and technical physician from the eHealth team lead the instruction session, which was used to capture the baseline asthma status and to instruct the child and parents on the home-monitoring tools. A key factor in the success was to create a personal connection between the HCPs and the patient and to assess the individual goal of the child and parents, which they want to achieve during the eHealth care period. HCPs indicate that they often compared the asthma status during the eHealth period with the baseline status and the predefined goals of the patient.

*Unsatisfactory start of eHealth*: Despite the fact that the inclusion sessions were extensive and participants also received the instruction materials to take home, the HCPs noticed start-up problems in approximately half of the participants. For example, trouble logging in, not able to synchronize the smart inhaler or non-compliance by thoughtlessness. The HCPs tried to solve these problems via chat with instructions and instruction videos. However, this was not always successful, which resulted in three additional appointments. As the program progressed the HCPs adapted the baseline sessions; participants were asked to bring their phone so that the log-in and synchronization could be tested. Furthermore, there was agreed upon a fixed moment in the week to share a weekly update. The start-up problems reduced, but non-compliance remained hard to tackle. HCPs and participants indicated that pop-up reminders could help to achieve a structural routine within the daily life.

*Safe health care*: HCPs aimed to create a safety net at home with eHealth monitoring to prevent asthma exacerbations. In consult with the patient and based on the monitored data HCPs provide substantiated medical advises. Gradually, children and parents were picking up these advises and were building self-confidence towards safely managing their asthma on their own. This process however stands or falls on sufficient longitudinal data and interaction with the patient. To be able to provide safe care as replacement for hospital and outpatient care, the HCPs indicated that patients have to understand their own responsibility in eHealth care to inform the HCPs. For this purpose non-compliance flowcharts were created, discussed and agreed on with participants to guide when and how to remind for non-compliance. They also provided an eventual endpoint of exclusion if non-compliance remained, repeated itself or the participants did want to cease eHealth care. HCPs felt supported by the use of the flowcharts, as the transparency lead to an increased understanding of patients of their own responsibility.

*Symptomatic periods*: Participants were instructed to follow the regular way of care during acute situations and out of office times. However, during the program HCPs noticed that participants were often very uncertain about their symptoms: are they real? how severe are they? and does the reliever medication help? This is why the HCPs constructed and updated a set of standardized questions and actions (i.e. daily lung function pre and post reliever medication) in case dyspnea symptoms are reported. Depending on the results of this initial check-up, monitoring could be intensified and/or action plans started. The HCPs indicated that by using such a protocol essential symptomatic information is gathered, which gave them clarity on how to act in acute situations with the use of the available eHealth tools. HCPs also explicitly stated that during these symptomatic periods eHealth monitoring makes the most difference, as substantiated decisions could be made to prevent further exacerbation and ED visits.

*Therapy compliance*: Through smart inhalers we have been able to monitor and adjust therapy compliance and inhalation technology. Therapy compliance is the cornerstone of asthma management and therefore crucial information for HCPs. They indicated that knowing the actual therapy compliance led to a more transparent conversation with children/parents, discussing the barriers that play a role for them. Simultaneously HCPs were able to work together with patients (“shared-care”) towards practical solutions, such as embedding the intakes in the daily ritual of the children. Additionally, the smart inhalers enabled HCPs to monitor the progress and encourage improvements, which makes eHealth care extremely suitable for improving therapy compliance. HCPs also indicated from experience that transparent therapy compliance monitoring should be introduced as soon as possible from the start of treatment, as this period provides a window of opportunity in which patients feel reinforced instead of controlled by the HCPs.

*Inhalation technique*: The fact that we observed the inhalation technique (inhalation flow, duration and orientation) remotely with smart inhalers is unique and gave HCPs tools to gain insight into the actual dose of medicine that reached the lungs. It also enabled them to select the most appropriate inhaler type for the individual patient. Although this smart inhaler is extremely suitable for the use in pediatric eHealth asthma care, the HCPs reported to still experience nuisance from the technological development to the smart inhalers (new portal versions, only android compatibility, etc) at the start of the project. However, they also reported that during the project the functionality improved notably and they do want to continue monitoring with smart inhalers as it provided an objective measure that can be discussed directly and steer towards targeted advises on inhalation technique.

*Implementation in the workflow*: The HCPs were positive towards the implementation of eHealth care in their clinic. They indicated to prefer to make well substantiated medical decisions based on home measurement and real time communication with patients during symptoms. The HCPs had a weekly meeting with the eHealth team, which was experienced as pleasant and effective by the HCP’s as it ensured that every patient is screened weekly with all available data and communication that had come in. Furthermore, this meeting was used by the HCPs to divide the care tasks among themselves based on each other’s specialism. HCPs also experienced that it was sometimes difficult to plan the care tasks next to their other work-related activities as it is difficult to predict a realistic time-plan for eHealth care. By maintaining contact through the chat and if necessary expand with telephone consultations HCPs tried to prevent deterioration. However, if the situation deteriorated further scaling up was required and more time investments are needed to increase monitoring, consult with the pediatricians and execute the therapy plan. This can happen at every moment of the day/night during the week or even in the weekend. Despite the fact that patients were informed and did understand that if acute situations happen outside working times they had to follow regular care pathways, HCPs themselves reported that they often invested much time outside working hours to be able to conclude eHealth care till they could be reassured that the children were doing well or till the children were referred to the ED if they did not recover. Therefore, HCPs advise to really plan a working climate with shifts on-call, tasks per shift and the availability of a (pulmonary) pediatrician to consult with when needed.

*Target population*: HCPs experienced that patients with a high medical consumption, uncontrolled asthma and/or a poor symptom perception, based on the discrepancy between objective measurements and perceived symptoms, benefit the most from the personalized eHealth approach in terms of gaining self-management. HCPs point out that these children and parents were intrinsically motivated to be compliant to home-monitoring and learn to rely on these assessed measurements and signs.

*Duration of eHealth care*: HCPs experienced that eHealth asthma care helped to identify the prevalent treatment issues faster, which enabled HCPs and children/parents to reach a learning effect more quickly compared to regular outpatient follow-up. In this proof of concept study we included patients for a period of six months, but the question “what is a good period for eHealth care?” is still open. What is the right moment to include and exclude? and which data can help to determine this? HCPs basically indicate that exclusion should follow after stabilization of asthma control. However, the risk of exclusion at a relatively good time is falling into a gap afterwards. HCPs indicated that approximately five participants were getting into trouble again after exclusion from this eHealth care program. On the other hand, HCPs also saw that more than half of the participants were able to achieve a stable situation well within the period of six months, which also remained after the end of the study. This emphasizes the real challenge of identifying those with whom HCPs can de-escalate long-term care. All HCPs indicated that it is paramount for eHealth care that it can be easily restarted and that follow up research should focus on identifying the children that got enough "luggage" for the future to be able to control their asthma versus the children that are still at risk for exacerbations. Additionally, eHealth care protocols should be made adaptive to the individual needs of a patient (i.e. trimmed eHealth care with specific device selection or intermittent monitoring only during symptomatic periods) to prevent an overkill of monitoring but allowing easy to access contact with the HCPs.

*The ideal eHealth asthma care for HCPs*:

When HCPs were asked to elaborate about their vision on the ideal eHealth care they often first mention: “speed-up the development of eHealth technology”. Many potential improvements were mentioned such as graphical representation of the most important data so that trends can easily be observed, automatic pop-ups for deviating values ​​from the portal, the possibility to set actions/reminders for the participants and last but not least the option to link data from the eHealth system to the electronic patient record. According to the HCPs these practical improvements are expected to greatly improve the current system will ensure that eHealth care can be performed more accurately and efficiently. In addition to technological development HCPs have already experimented with the distribution of care tasks and the planning of eHealth care (task reallocation). Ideally, after scaling up, the portal should be checked 24/7 by an asthma nurse and there should always be the possibility of stepwise consultation and scaling up to; step 1: Nurse practitioner or technical physician and step 2: (pulmonary) pediatrician, so that eHealth care is always properly guaranteed. In addition, it is preferred to schedule fixed shifts for HCPs. To make this possible, upscaling is desirable and regional cooperation with other hospitals could be considered.
